# Anatomic Distribution of Nerves and Microvascular Density in the Human Anterior Vaginal Wall: Prospective Study

**DOI:** 10.1371/journal.pone.0110239

**Published:** 2014-11-07

**Authors:** Ting Li, Qinping Liao, Hong Zhang, Xuelian Gao, Xueying Li, Miao Zhang

**Affiliations:** 1 Department of Obstetrics and Gynecology, Peking University First Hospital, Beijing, China; 2 Department of Pathology, Peking University First Hospital, Beijing, China; 3 Department of Statistics, Peking University First Hospital, Beijing, China; University of Sydney, Australia

## Abstract

**Background:**

The presence of the G-spot (an assumed erotic sensitive area in the anterior wall of the vagina) remains controversial. We explored the histomorphological basis of the G-spot.

**Methods:**

Biopsies were drawn from a 12 o’clock direction in the distal- and proximal-third areas of the anterior vagina of 32 Chinese subjects. The total number of protein gene product 9.5–immunoreactive nerves and smooth muscle actin–immunoreactive blood vessels in each specimen was quantified using the avidin-biotin-peroxidase assay.

**Results:**

Vaginal innervation was observed in the lamina propria and muscle layer of the anterior vaginal wall. The distal-third of the anterior vaginal wall had significantly richer small-nerve-fiber innervation in the lamina propria than the proximal-third (p = 0.000) and in the vaginal muscle layer (p = 0.006). There were abundant microvessels in the lamina propria and muscle layer, but no small vessels in the lamina propria and few in the muscle layer. Significant differences were noted in the number of microvessels when comparing the distal- with proximal-third parts in the lamina propria (p = 0.046) and muscle layer (p = 0.002).

**Conclusions:**

Significantly increased density of nerves and microvessels in the distal-third of the anterior vaginal wall could be the histomorphological basis of the G-spot. Distal anterior vaginal repair could disrupt the normal anatomy, neurovascular supply and function of the G-spot, and cause sexual dysfunction.

## Introduction

The “Gräfenberg spot” (“G-spot”) was named by John Perry and Beverly Whipple [1] in reference to the pioneering work of Dr. Ernst Gräfenberg in 1950 [2]. The G-spot as a distinct erotogenic zone on or under the anterior vaginal wall about halfway between the back of the pubic bone and the cervix, along the course of the urethra, has been accepted by many sexologists and women. However, there are conflicting data about the existence of the G-spot in the vagina [3]–[12]. Defined as a “modern gynecologic myth”, the hypothesis that the G-spot is located ≈1 cm from the surface of the vaginal anterior wall and one-third to one-half the way from the vaginal opening is weakly supported by behavioral, anatomical, and biochemical evidence [9], [13]–[15]. Thabet [16] confirmed that the G-spot was actually present in all women, localized spot in 58% and diffuse in 42% of cases. A questionnaire study in the USA and Canada showed that most women could feel and locate their G-spot [4]. In a large study investigating the underlying genetic basis of the G-spot, Burri et al. found a lack of heritability of the ability by women to detect their own G-spot [14]. They postulated that the reason for the lack of genetic variation was that there was no physiological or physical proof for the G-spot [14]. Biochemical evidences for the G-spot came from analyses of female ejaculation fluid [2], [4], [17]. Puppo and Gruenwald hypothesized that the vasculature and dorsal nerve of the clitoris were not shared with the distal urethra and vagina [10]. However, studies yielding positive evidence involved only small participant samples and those yielding negative evidence showed methodological biases [5], [6], [18].

This modern gynecologic myth is far from being understood. However, most proponents agree that the G-spot comprises resembled cavernous erectile tissue and paraurethral (Skene’s) duct as the basic structure, with histological evidence of clitoris-shared vasculature and nerve distribution [11], [19]–[21]. Skene’s glands are located in the posterior portion of the female urethra and the anterior portion of the vagina. Skene’s glands are homologous to the male prostate gland in terms of eliciting orgasmic ejaculation. Thus, several researchers consider the G-spot to be the “female prostate gland” [2], [4], [11], [17], [22].

Only a few G-spot’s studies can be relevant to the anterior wall, especially quantifying vaginal nerve fibers. In 1995, Hilliges et al. found that not only was the anterior wall (in general) more dense than the posterior wall, but also that the distal area had more nerve fibers compared with the proximal part [5]. In another study, the second one-fifth partition of the distal anterior wall had significantly richer innervation than the surrounding areas, and was thought to be the “hypothetical G-spot” [6]. However, a study of the biopsy specimens of 21 patients’ by Pauls et al. argued that vaginal nerves were located regularly without a specific vaginal locus with increased nerve density [18]. With regard to the vasculature, few scholars have focused on the network of microvessels in the vaginal wall.

The aim of the present study was to explore the total anatomical distribution of nerves and microvascular density in the anterior vaginal wall of adult humans.

## Materials and Methods

### Ethical approval of the study protocol

The study protocol was approved by the Ethics Committee of Peking University First Hospital (Beijing, China), and our study has been conducted according to the principles expressed in the Declaration of Helsinki. All eligible participants provided written informed consent to be included in this study.

### Participants

This was a prospective study of patients undergoing vaginal surgery for prolapse and incontinence at Peking University First Hospital between November 2010 and July 2012. Exclusion criteria comprised a history of diabetes mellitus, multiple sclerosis, peripheral neuropathy or any other disease that could affect the distribution of nerves and small vessels. Thirty-two women (mean age, 60.5±9.8 years; range, 41–77 years) agreed to participate in this study. Twenty-two in twenty-four postmenopausal women have applied short-term topical vaginal Estriol cream (0.5 g/d) before the date of surgery. Information relating to sexual practices and sexual history was not obtained.

### Specimen collection

Specimens were obtained in a standardized manner by one investigator. Six biopsies were obtained from patients with stress urinary incontinence undergoing tension-free vaginal tape surgery. Twenty-six full-thickness specimens were dissected from the prolapsed anterior vaginal wall of each patient with pelvic organ prolapse (9 cases of mesh repair and 17 cases of conventional pelvic-floor surgery). During colporrhaphy, the entire length of the vagina was measured. The distal-third and proximal-third areas of the anterior vagina wall at a 12 o’ clock direction were marked. Specimens were harvested from these areas perpendicular to the longitudinal axis after the incisions for colporrhaphy were made.

### Immunohistochemical analyses

All biopsies were fixed in 10% neutral buffered formalin and embedded in paraffin using conventional histopathological methods. Paraffin blocks were serially dissected at a thickness of 5 µm with ten histological sections preserved per glass slide. Each section was stained with hematoxylin and eosin (H&E) for routine microscopy, general neural marker protein gene product 9.5 (PGP9.5, Dako, Glostrup, Denmark) as well as a marker of vascular smooth muscle, smooth muscle actin (SMA; Dako), following the avidin-biotin-peroxidase procedure using an EnVision Automated Immunostainer (Dako). As negative controls, sections were treated in an identical manner but with 0.01 M phosphate-buffered saline replacing the primary antibody. All blind-coded sections were evaluated independently by two experienced pathologists and examined under a double-headed microscope (BH2; Olympus, Tokyo, Japan). Dropsical tissue would be excluded.

The exact numbers of nerves and vessels were manually quantified in five consecutive fields under high power (×40). Blood vessels were classified in four categories according to diameter: small artery (0.3–1 mm), small vein (≥200 µm), arteriole (<300 µm) and venule (<200 µm) [23]. Incomplete blood vessels on the right edge were quantified and those on the left were ignored.

### Statistical analyses

Statistical analyses were conducted using SPSS ver13.0 (SPSS, Chicago, IL,USA). Differences in PGP9.5-immunoreactive (PGP9.5-ir) nerve fibers and SMA-immunoreactive (SMA-ir) blood-vessel densities were tested for significance by paired-sample or independent-sample *t*-tests. p<0.05 was considered significant.

## Results

Thirty-two women (mean age, 60.5±9.8 years; range, 41–77 years) agreed to participate in this study. No sample was excluded due to tissue edema after H&E staining. Eight participants were premenopausal women with normal monthly menstrual cycles, and 24 were postmenopausal women.

### Nerves

**Figure 1:**
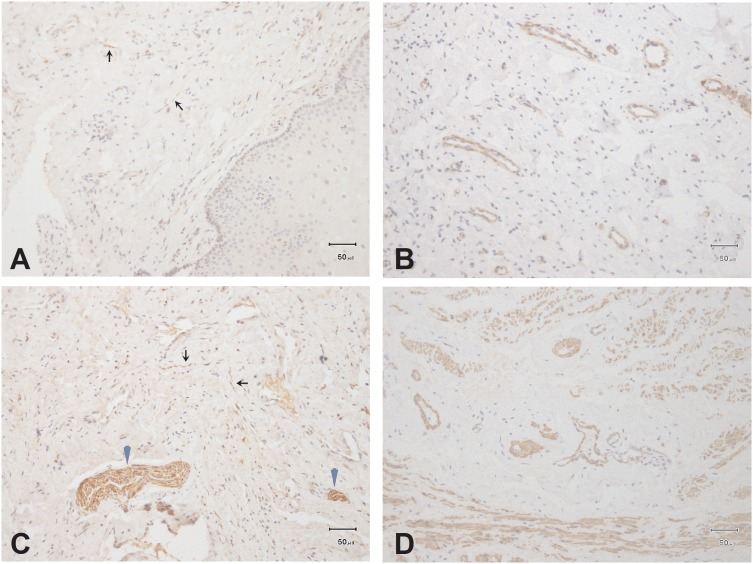
PGP9.5 immunostaining: Vaginal tissue sections from the lamina propria layer (A) and muscle layer (C), nerve bundles indicated by blue arrowheads, small nerves indicated by small black arrows. **SMA immunostaining:** Vaginal tissue sections from the lamina propria layer (B) and muscle layer (D). Scale bar represents 100 mm (200×magnification).

Nerve fibers were observed in all specimens, but not in the vaginal epithelium. Small nerve fibers were detected in the lamina propria and muscle layers of distal- and proximal-third areas. Nerve bundles were less abundant in the muscle layer and rarely appeared in the lamina propria. Differences between distal-third and proximal-third areas are presented in Figure 1.

The distal-third areas of the anterior vaginal wall were significantly richer in small nerve fibers in the lamina propria and muscle than proximal-third areas (p = 0.000 and 0.006) (Table 1).

**Table 1:** Vaginal innervation according to location (alpha = 0.05, two-tailed).

Location	Subject N	Nervetype	Distal-third(per mm^2^)	Proximal-third(per mm^2^)	*P*
Lamina propria	32	Fibers	4.34±0.35	2.34±0.29	0.000*
Muscle layer	31†	Fibers	5.23±0.49	3.61±0.33	0.006*
		Bundles	2.16±0.49	1.35±0.31	0.085

*p<0.05 (paired samples *t*-tests);

† one specimen did not contain a muscle layer due to superficial surgical biopsy.

### Blood vessels

There was abundant microvasculature in the lamina propria and muscle layer of the anterior vaginal wall. However, no small vessels in the lamina propria and few in the muscle layer were found. Differences between distal- and proximal-third areas are detailed in Figure 1.

Significant differences were noted in the number of microvessels in the distal-third compared with the proximal-third areas of the anterior vaginal wall in lamina propria and muscle layer (p = 0.046 and 0.002) (Table 2).

**Table 2:** Quantification of vaginal vessels according to location (alpha = 0.05, two-tailed).

Location	Subject N	Vessel type	Distal third(per mm^2^)	Proximalthird (per mm^2^)	*P*
Lamina propria	32	Micro	54.25±3.07	47.88±2.20	0.046*
Musclelayer	31†	Micro	28.61±2.03	24.71±1.99	0.002*
		Small	0.23±0.08	0.19±0.07	0.748

*p<0.05 (paired-sample *t*-test); Micro, microvessels; small, small vessels;

† one specimen did not contain a muscle layer due to superficial surgical biopsy.

### Premenopausal group and postmenopausal group

The quantity and distribution of nerves and vessels in premenopausal and post-menopausal groups were examined (Table 3). Irrespective of whether in the lamina propria or muscle layer of distal- and proximal-third areas, there were significant differences in terms of fibers and microvessels based in the postmenopausal group (p = 0.000 and 0.012; p = 0.000 and 0.000). There was no significant difference between the two areas in the premenopausal group group (p = 0.059 and 0.602; p = 0.403 and 0.818).

**Table 3:** Histological findings in the vaginas of premenopausal and postmenopausal subjects (alpha = 0.05, two-tailed).

Location	Vessel type	Premenopause (n = 8)			Postmenopause (n = 24)		
		Proximal-third(per mm^2^)	Distal-third(per mm^2^)	*P*	Proximal-third(per mm^2^)	Distal-third(per mm^2^)	*P*
**Lamina propria**	**Fibers**	3.12±0.515	5.12±0.718	0.059	2.08±0.329	4.08±0.394	0.000*
	**Microvessels**	47.25.±5.570	44.25±5.324	0.602	48.08.±2.332	57.56.±3.494	0.012*
**Muscle** **layer**	**Fibers**	5.14±0.595	6.14±0.634	0.403	3.17±0.344	4.96±0.596	0.000*
	**Microvessels**	22.86±2.262	23.57±3.531	0.818	25.25±2.484	30.08±2.357	0.000*

*p<0.05 (paired-sample *t*-test).

## Discussion

### Innervation of the G-spot

Only a few studies have focused on innervation of human vaginal wall, and none have focused on vaginal microvasculature. Moreover, the findings showing some differences in vaginal nerve distribution have not proven to be universally reproducible [12]. Hilliges et al. did not quantify the nerve endings or consider other factors influencing nerve distribution [5]. Pauls et al. used the specific marker S100 when staining myelinated nerve fibers rather than unmyelinated small nerve fibers (especially nociceptive C fibers) [18]. We used the immunohistochemical marker PGP9.5, which can stain all axons and nerve terminals in the skin and mucosa of humans. PGP9.5 has also been used for counting nerve fibers in the human vagina [24]. PGP9.5 and S100 are pan-neuronal markers, but PGP9.5 is a ubiquitin C-terminal hydrolase that permits visualization of the nerve network in the vagina irrespective of whether it is myelinated or unmyelinated [5], [8], [25]. Song et al. [6] postulated that the second one-fifth partition from the inferior anterior wall could be the G-spot. Nevertheless, for only 2 immunostained samples, there was no significant difference in the number of nerve fibers between distal and proximal parts. Overall, convincing neurovascular evidence supporting the existence of the G-spot is lacking.

The G-spot could be located within the anterior vaginal wall, one-third to one-half of the way from the introitus [20]. The G-spot in humans may have a feature of the copious number of nerves. Our findings confirmed that the distal-third areas of the anterior vaginal wall bore a significantly greater number of nerves in the lamina propria and muscle layer than proximal-third areas. The number of PGP9.5-ir small nerve fibers in the lamina propria was slightly fewer than empirical datum of Song et al. but the count in the muscle layer was higher. The proportion of bundles was very small compared with the proportion of small fibers, but there were more bundles in the distal-third wall (though the difference was not significant). The muscularis contained more nerve fibers than the lamina propria, findings that were similar to those of another investigator [26].

These neurobiological divergences can be attributed to the embryological origin of the distal-third and proximal-two-thirds 2/3 parts. The urogenital sinus and Müllerian ducts, respectively, lay the groundwork for the G-spot.

### Vascular distribution of the G-spot

Master and Johnson observed intravaginal changes through specific imaging equipment and discovered that vaginal vasocongestion occurred from the initial period of sexual excitation [27]. They noted that the tissue hyperemia experienced during orgasm was extraordinarily obvious in the distal-third part (“orgasm platform”). Several researchers believe that the G-spot is probably composed of a complex network of blood vessels.

Our study revealed more microvessels in the lamina propria and muscle layer of the distal-third areas of the anterior vaginal wall compared with proximal-third areas. Vascular engorgement of the vagina is the motor component of the female sexual reflex, and its associated lubrication can relieve dyspareunia. If vaginal microvessels (especially those from the circumvaginal veins) become congested, then edema fluid will pour into all the interstitial spaces and fat-filled compartments; hence, fluid that crosses the vaginal mucosa will become a vaginal lubricant [28]. The more microvessels that this region has, the more swollen it maybe become to accept tactile stimulation and reach vaginal-activated orgasm. A significant difference was observed in the number of microvessels in the present study, but further investigation is needed to ascertain whether such a difference could cause functional changes.

### Menopause and G-spot

We noted significant differences in the number of nerve fibers and microvessels between distal-third and proximal-third areas in women in the postmenopausal group, but not so in the premenopausal group. This may have been due to: (i) the small sample size or because most postmenopausal women underwent preoperative short-term estrogen treatment (which may have enabled regulation of hormone-sensitive nerves and vessels). Further studies should focus on whether hormonal–neural interactions occur in the human vagina to ascertain if estrogen exerts direct effects on the various types of vaginal nerves.

### G-spot and anterior vaginal repair

Different studies focusing on sexual function after pelvic reconstructive surgery have elicited different results. Sentilhes et al. used scores from the Lemack Questionnaire and Pelvic Organ Prolapse/Urinary Incontinence Sexual Questionnaire-12 to evaluate sexual function before and after transvaginal mesh repair of genital prolapse. They found that transvaginal mesh repair did not impair sexual function 1 year after surgery. They also found that, in women who reported diminished sexual function, this phenomenon could be (at least in part) the result of impaired nerve function [29]. Occhino et al. also believed that vaginal surgery would not cause changes in sexual function despite a shortened and narrowed vagina [30]. By contrast, several researchers deemed anterodistal vaginal dissection or repair of prolapsed vagina to not only injure cavernous nerves at the anterolateral side of the distal vagina (which contains clitoral tissue) [31], but may also damage nerve fibers of the G-spot within the lamina propria and muscle layer, and leads to difficulty in sexual arousal or achieving orgasm, dyspareunia, and negative emotional reactions after surgery [32]. Recently, Thabet [16], [20] revealed that the sexual scores were significantly lowest in the cases in which surgeries having similar operative G-spot involvement, including much dissection of the anterior vaginal wall, excision of parts of the anterior vaginal wall and the hymenal connecting areas. Research regarding the G-spot and female sexual dysfunction is in its initial stages, and many phenomena need to be investigated.

## Conclusion

A prospective study involving biopsies of the proximal-third and distal-third of the anterior vaginal wall was conducted. The distal-third has more innervations and better vascularization, so the vagina may have a sexual-sensitive function, just like the clitoris. Furthermore, our results suggest that excessive alloplastic materials or dissection of a prolapsed anterior vaginal wall could disrupt the normal anatomy, neurovascular supply, and function of the G-spot, and cause sexual dysfunction. The present study will help pelvic surgeons choose a G-spot-sparing route for vaginal surgery, especially in women with pelvic organ prolapse.
